# Predicting Energy Consumption Using LSTM, Multi-Layer GRU and Drop-GRU Neural Networks

**DOI:** 10.3390/s22114062

**Published:** 2022-05-27

**Authors:** Sameh Mahjoub, Larbi Chrifi-Alaoui, Bruno Marhic, Laurent Delahoche

**Affiliations:** 1 Laboratory of Innovative Technology (LTI, UR 3899), University of Picardie Jules Verne, 80000 Amiens, France; larbi.alaoui@u-picardie.fr (L.C.-A.); bruno.marhic@u-picardie.fr (B.M.); laurent.delahoche@u-picardie.fr (L.D.); 2 Control & Energy Management Laboratory (CEMLab), University of Sfax, Sfax 3029, Tunisia

**Keywords:** neural networks, time series, LSTM, GRU, Drop-GRU, energy consumption prediction, load shedding

## Abstract

With the steep rise in the development of smart grids and the current advancement in developing measuring infrastructure, short term power consumption forecasting has recently gained increasing attention. In fact, the prediction of future power loads turns out to be a key issue to avoid energy wastage and to build effective power management strategies. Furthermore, energy consumption information can be considered historical time series data that are required to extract all meaningful knowledge and then forecast the future consumption. In this work, we aim to model and to compare three different machine learning algorithms in making a time series power forecast. The proposed models are the Long Short-Term Memory (LSTM), the Gated Recurrent Unit (GRU) and the Drop-GRU. We are going to use the power consumption data as our time series dataset and make predictions accordingly. The LSTM neural network has been favored in this work to predict the future load consumption and prevent consumption peaks. To provide a comprehensive evaluation of this method, we have performed several experiments using real data power consumption in some French cities. Experimental results on various time horizons show that the LSTM model produces a better result than the GRU and the Drop-GRU forecasting methods. There are fewer prediction errors and its precision is finer. Therefore, these predictions based on the LSTM method will allow us to make decisions in advance and trigger load shedding in cases where consumption exceeds the authorized threshold. This will have a significant impact on planning the power quality and the maintenance of power equipment.

## 1. Introduction

Power consumption forecasting has been considered as a key challenging topic for smart grid planning, electricity market development, and the sustainability of power. Recent research shows that precise power prediction provides important guidance to the power suppliers and consumers to improve power management, secure the grid and control the load [1]. So, with the recent development of sophisticated machine learning-based methods and especially deep learning algorithms, a large number of researchers in diverse disciplines have focused on these techniques [2]. Thus, time series analysis has become a popular research area. Its development has a great impact on our daily life [3]. It can be used to track how a sensor value, economic variable or weather report changes over time. It is also applied everywhere to forecast future outcomes based on recorded historical data in several fields such as weather prediction [4], air pollution prediction [5] and stock market prediction [6]. In this context, many prediction methods have been proposed in the literature. From an academic point of view, these methods can be divided into three categories: statistical analysis, machine learning and deep learning. The statistical models include various methods such as Markov chain (MC) [7], exponential smoothing [8] and autoregressive integrated moving average (ARIMA) [9]. The machine learning models consist of three methodologies: Decision Tree (DT), Support Vector Machine (SVM) and Artificial Neural Network (ANN) [10]. Finally, as a breakthrough in artificial intelligence, deep learning approaches automatically perform in treating highly nonlinear features via a cascade of multiple layers. Recurrent Neural Network (RNN) and Back-Propagation Neural Network (BPNN) are two popular algorithms used for predicting time series [11]. So, a forecasting system based BPNN is widely used by researchers, due to its strong adaptability and computing ability. However, it also has some shortcomings, such as the final training result which can easily fall into a local extremum [12]. RNN can remember the historical information and choose whether to retain this information for the current output. Nevertheless, it fails to maintain the long temporal dependence because of the serious vanishing/exploding gradient problem [13]. To alleviate these problems, an improved version of RNN, named long short-term memory network (LSTM) is proposed. The LSTM network is based on the introduction of a cell memory in the RNN which presents a promising solution to inhibit the gradient disappearance [14]. Their contribution is particularly manifest in the case of long sequences of events. It has also demonstrated a significant improvement in terms of forecasting stability. The LSTM can give more stable forecasting power in time-series prediction compared to traditional RNN [15]. In this paper, an LSTM network based algorithm for forecasting power consumption is presented. Indeed, LSTM models are currently widely used as the most robust approach to dealing with sequential data. Their contribution is especially obvious in the case of rather long sequences of events. Time series analysis and forecasting are currently at the beginning of their potential. It should be noted that, in the case of the construction of global models for the time series, the LSTM models allow the creation of high-performance models, both for point forecasts and long-term forecasts [16]. The fundamental question is then how powerful and accurate these newly introduced techniques are when compared with traditional approaches. In this work, a dynamic model based on time series analysis and the LSTM is proposed to forecast the power consumption, detect the power peaks, and then shed the load. The aim of this prediction is to maintain the power balance between producers and consumers to ensure the security of the electrical grid. In order to make full use of the various data in the power consumption and achieve accurate predictions, different deep learning-based algorithms were proposed, which comprise the LSTM, the GRU, and the Drop-GRU models. In the proposed model, the GRU module is exploited to model dynamic changes in historical power consumption data for better learning potential characteristics in time sequence data, and the dropout process is used as a weight regularization technique for reducing overfitting. In an analogous way, the LSTM method is chosen due to its ability to preserve and train the characteristics of the provided data for a long period of time. Therefore, this technique has achieved a significant success in recent years. Indeed, the paper provides in-depth guidance on data processing and training of LSTM models for a set of power consumption time series data. Moreover, a comparison study between GRU, Drop-GRU, and LSTM models concerning their performance in reducing error rates is performed. The article is organized as follows. Section 2 presents the context of this work. Section 3 explains the methodology adopted in this study. Firstly, it provides the general framework of the proposed predictive models. Then it gives, step by step, the implementation process of the proposed algorithms; it covers the dataset processing description, the parameters and the evaluation indices. Section 4 shows the experimental detail, the results analysis and the performance evaluation of the LSTM networks in comparison to the GRU and the Drop-GRU models. Finally, Section 5 concludes this work.

## 2. Context of This Work

**Figure 1:**
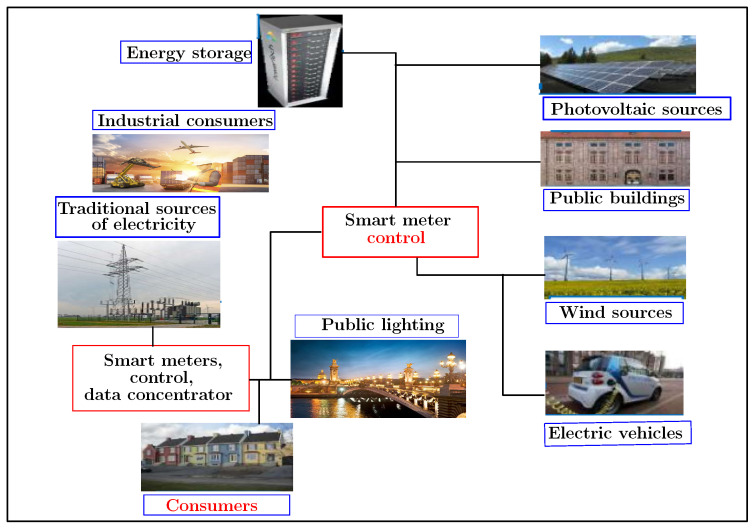
The smart grid context: VERTPOM project.

The working subject presented in this manuscript falls within the framework of the development of a monitoring platform related to energy management on the consumer side. It also aims to improve the energy efficiency of public infrastructure and optimize the electrical networks. Figure 1 illustrates the general context of this project. Indeed, this subject is part of a vast VERTPOM research project which wants to develop positive energy territories through the design of smart electricity grids. The VERTPOM project will maintain an optimized balance between the energy available from production regarding uses by applying a set of algorithms for forecasting and simulating the levels of power production and consumption on the various distribution systems. It treats an actual database. The use of artificial intelligence networks, such as machine and deep learning, is preferred. The key challenge of this project is to simulate all possible scenarios allowing a positive production/consumption balance. Hence, the main objectives of VERTPOM are anticipating the power requirement according to all the parameters available in the area such as climatology, scheduled events and consumption in order to make the best decision regarding consumption priorities among renewable and conventional energies. Moreover, it should optimize the peak consumption and interact intelligently with the consumer-player. So the aim of all previous objectives is to develop a set of digital tools for power management, to design adequate smartgrids for energy suppliers and consumers and to guarantee the safety of electrical infrastructures.

## 3. Materials and Methods

**Figure 2:**
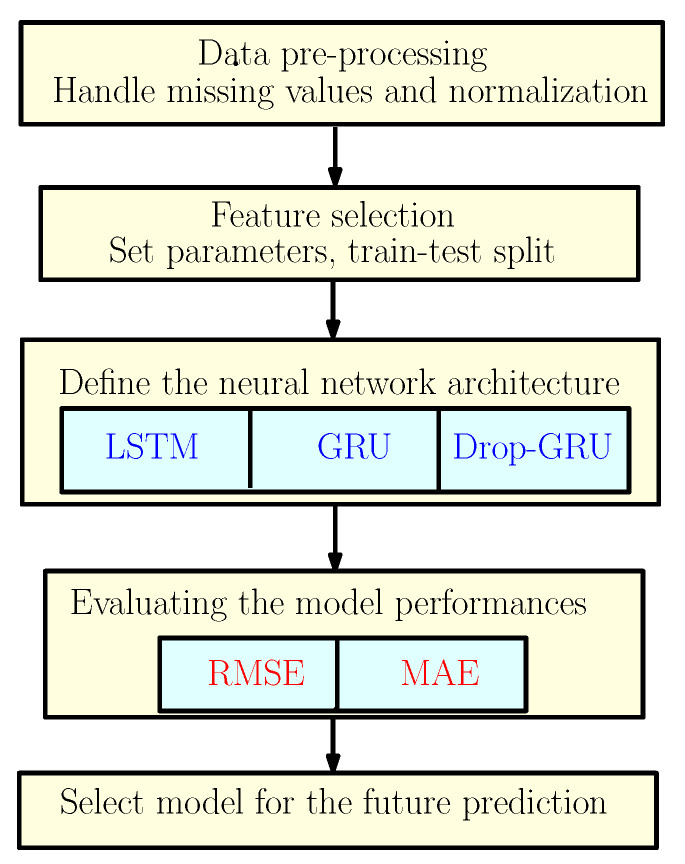
The Proposed Methodology.

To predict the energy consumption, we suggest a framework based on three neural network models as discussed earlier. So to explain the methodology of our work, the flowchart shown in Figure 2 gives different steps used to construct the proposed predictive models.

Before discussing the forecast, we should understand how important analysing and preprocessing data are for time series. They can make or break the forecast. Thus, the main challenge to forecasting is preprocessing data into the appropriate form.

### 3.1. Data Analysis

The processed data used in this work represent the average power consumption of an area in Péronne city. There are several zones; a measurement unit in each zone allows us to record the energy consumption data. These data are logged by measurement tools from the VERPOM team with a sampling rate of 30 min.

The measured values depend on several factors:

- Nature of consumers: inhabitants, factories, hospitals, offices, etc.;
- Climatology: humidity, temperatures, sunshine, wind speed, etc.;
- Days of the week, weekends, holidays, etc.

**Figure 3:**
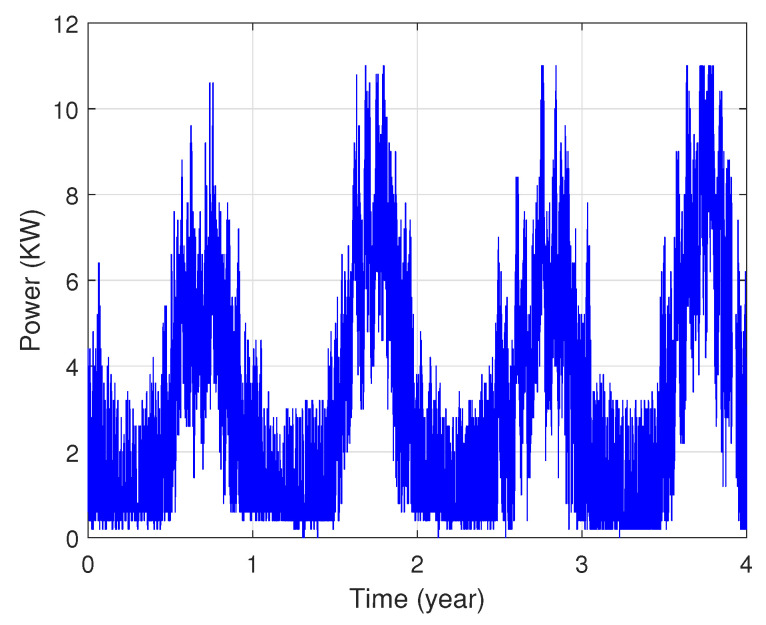
Power consumption of the first zone.

**Figure 4:**
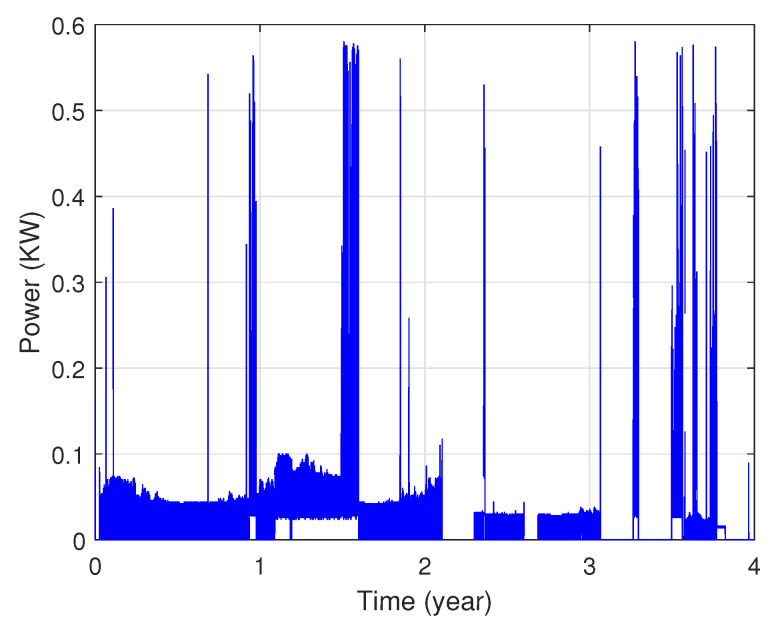
Power consumption of the second zone.

In fact, these data show that each area’s power consumption has various distribution characteristics as illustrated in Figure 3 and Figure 4. The presented profiles are of different types: cyclical, irregular, stationary and with trends, etc.

### 3.2. Data Pre-Processing

Data tend to be incomplete and incoherent, therefore data cleaning aims to remove noise, fill in missing values and correct inconsistencies in the data. In this section, we will present the crucial preprocessing steps for time series. The aims of standardization is that the model runs very fast and to bring values to specific ranges. Hence, data preprocessing involves various operations. Each operation aims to help the machine learning to develop the best predictive models:

- Some raw data have “holes”: the process of Exponential Moving Average (EMA) is used to fill in the missing information.
  The EMA is a type of moving average that gives a greater weight and significance to the most recent data points. The *EMA* formula is given as:
  (1) $$EMA=\left(Value_{today}-{EMA}_{yesterday}\right)*Smoothing\;constant+{EMA}_{yesterday},$$
  where the smoothing constant is equal to $\frac{2}{n+1}$
  with *n* as the number of time periods.
  The EMA formula is based on the previous day’s EMA value. Since it has to start the computations somewhere, the initial value for the first EMA calculation will actually be an SMA. It is calculated by taking the arithmetic mean of a given set of values over a specified period of time.
  The formula for computing the *SMA* is presented as:
  (2) $$SMA=\frac{A_{1}+A_{2}+\ldots +A_{n}}{n},$$
  where
  *A* denotes the average in period *n*.
- Consumption profiles have different types of data: the process of reduced centered standardization is applied. The normalisation is performed by $Z_{Score}$ and the formula is given as:
  (3) $$Z_{Score}=\frac{x-x_{mean}}{x_{\sigma }},$$
  where:
  (4) $$x_{\sigma }=\sqrt{\frac{1}{n-1}\sum_{i=1}^{n}{\left(x_{i}-x_{mean}\right)}^{2}}$$
  (5) $$x_{mean}=\frac{1}{n}\sum_{i=1}^{n}x_{i}.$$

The goal of data standardization is to use a common scale, without loss of information. The idea is to push all the values of the variable to be between −1 and 1, while keeping the distances between the values. These two preliminary preprocessing steps allow the LSTM network to work properly.

### 3.3. LSTM Neural Network Model

**Figure 5:**
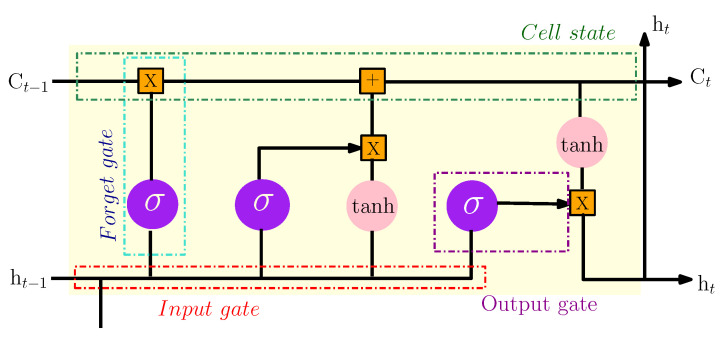
The internal structure of the LSTM model.

The long short term memory (LSTM) model is a special form of the recurrent neural network (RNN). This model conserves long-term memory by using memory units that can update the previous hidden state. It provides feedback at each neuron. The output of RNN is not only dependent on the current neuron input and weight but also dependent on previous neuron inputs. This functionality makes it possible to understand temporal relationships on a long-term sequence. Its internal memory unit and gate mechanism overcome the exploding and vanishing gradient problems that occur in training traditional RNN. So, the internal structure of the LSTM model includes four important units: input gate, output gate, forget gate and cell status. The three gates are introduced to control the maintenance and the update of information comprised in cell status. Figure 5 shows the structure of an LSTM cell. The computation process can be given in the following as [17]:

(6) $$f_{t}=\sigma \left(w_{f}\left[h_{t-1},X_{t}\right]+b_{f}\right)$$

(7) $$i_{t}=\sigma \left(w_{i}\left[h_{t-1},X_{t}\right]+b_{i}\right)$$

(8) $$o_{t}=\sigma \left(w_{o}\left[h_{t-1},X_{t}\right]+b_{o}\right)$$

(9) $$a_{t}=tanh\left(w_{a}\left[h_{t-1},X_{t}\right]+b_{a}\right)$$

(10) $$c_{t}=f_{t}*c_{t-1}+i_{t}*a_{t}$$

(11) $$h_{t}=o_{t}*tanh\left(c_{t}\right),$$

where σ is the sigmoid activation function and it can be defined as:

(12) $$\sigma \left(x\right)={\left(1+e^{-x}\right)}^{-1}.$$

The notations $f_{t}$, $i_{t}$ and $o_{t}$ are the output values of the forget, the input and the output gates, respectively. $c_{t}$ refers to the memory cell and $a_{t}$ is the update and the activation of the current cell status. $X_{t}$ is the input vector and $h_{t}$ represents the output vector result at time *t*. Finally, $W_{f,i,a,o}$ are the weights matrices and $b_{f,i,a,o}$ the bias vectors.

**Figure 6:**
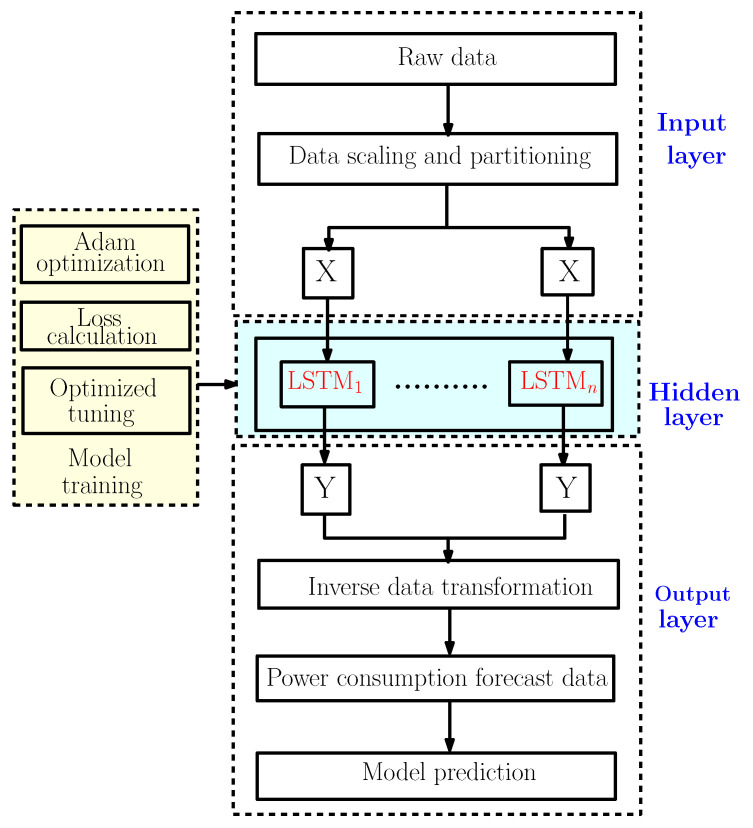
The LSTM power consumption forecasting framework.

Figure 6 illustrates the forecast strategy framework with the LSTM model. This model can be divided into three big parts [18]:

- The input layer is mainly used for preprocessing the original data;
- The hidden layer is used to optimize the parameters and training the data;
- The output layer is used to predict the data according to the model trained in the hidden layer.

### 3.4. LSTM Network Parameters

The complexity of the network is characterized by its trainable parameters of the network, which are called the trainable weights. They are illustrated through the connections between the input layer, the hidden layer and the output layer as well as internal connections in LSTMs. For a neural network of *n* inputs, *m* outputs and *p* LSTM cells in the hidden layer, the Number of Trainable Weights (*NTW*) is calculated as:

(13) NTW=4np+4pp+4p+mp+m.

Table 1 explains each parameter used in the previous equation.

Selecting optimal parameters for a neural network architecture can often mean the difference between poor and peak performances. However, there is little information in the literature on the choice of different parameters, *n*, *m* and *p*, of the neural network; it involves the experience of experts.

### 3.5. GRU Neural Network Model

**Figure 7:**
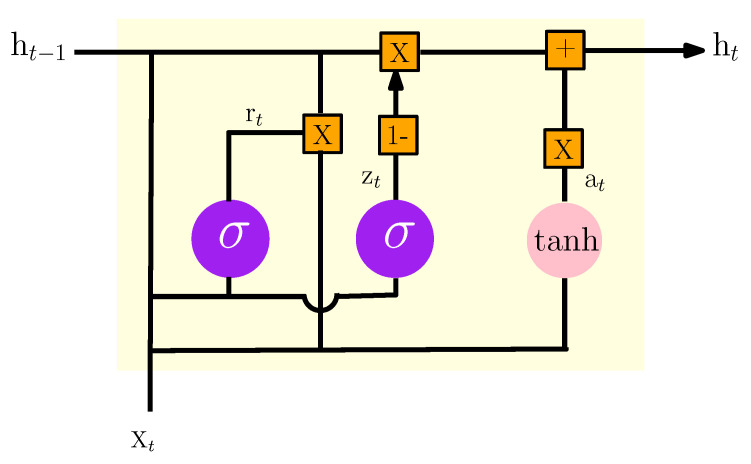
The internal structure of the GRU model.

GRU is one of the most popular improved variants of RNN with a special gated recurrent neural network based on optimized LSTM. The GRU internal structure is similar to the internal structure of the LSTM, except that the GRU associates the input gate and the forget gate in the LSTM unit into a single update gate. This model has two gates: one is the update gate, which controls the extent and retains previous information in the current state; the other represents the reset gate which determines whether the previous information and the current state are to be associated [19]. Figure 7 shows the basic design of a GRU unit.

According to Figure 7, the formulas of GRU can be given as:

(14) $$z_{t}=\sigma \left(w_{z}\left[h_{t-1},X_{t}\right]+b_{z}\right)$$

(15) $$r_{t}=\sigma \left(w_{r}\left[h_{t-1},X_{t}\right]+b_{r}\right)$$

(16) $$a_{t}=tanh\left(r_{t}*w_{a}\left[h_{t-1},X_{t}\right]+b_{a}\right)$$

(17) $$h_{t}=\left(1-z_{t}\right)*a_{t}+z_{t}*h_{t-1},$$

where $X_{t}$ is the vector input of training data at time *t*, and $h_{t}$ is the outcome of the current layer at time *t*. $z_{t}$ and $r_{t}$ represent the update and the reset gates respectively. $a_{t}$ is the candidate activation.

### 3.6. Neural Network Model Setup

#### 3.6.1. Gradient Descent Algorithm

In order to address an optimization problem, an energy function based on the bound constraints is defined. The traditional gradient descent is an effective algorithm for constrained optimization problems which can be used to minimize the energy. It needs extensive time and computing resources to converge when training the neural network. The optimizer is exploited to adjust the weight and offset of the model so that the algorithm converges rapidly to the optimal value and then loss can be minimized [20]. Considering *E* is an energy function of *n* variables $u_{1}$ to $u_{2}$ [21]:

(18) $$E\left(u\right)=E\left(u_{1},u_{2},\ldots ,u_{n}\right).$$

The gradient vector can be given as:

(19) $$\frac{\partial E}{\partial u}=\left(\frac{\partial E}{\partial u_{1}},\frac{\partial E}{\partial u_{2}},\ldots ,\frac{\partial E}{\partial u_{n}}\right).$$

To converge to the minimum of *E*, if each element of the gradient vector is negative then the variable of this element is increased. However, if each element of this vector is positive, then the corresponding variable is decreased. Using this technique, the recursive equation, to update the variables, is established as:

(20) $$u_{i+1}=u_{i}-\alpha \frac{\partial E}{\partial u_{i}}\;\Rightarrow ▵u_{i}=-\alpha \frac{\partial E}{\partial u_{i}},$$

where α represents the learning rate. It is a positive parameter. $u_{i}$ gives the value of variables in the ith iteration of running the algorithm. In this work, the Adam algorithm is chosen as the suitable optimizer that can update the network weights and improve the performances of our model. This algorithm needs less memory and is well adapted to solving problems that implicate the learning of complex and large datasets.

#### 3.6.2. Dropout

The deep learning neural network has a powerful memory. However, the network tends to learn the features of data that cannot be generalized, resulting in overfitting. Dropout is one of the most popular regularization techniques that was proposed to solve this problem. It returns the output of a proportion of the hidden units to zero randomly according to the Dropout in order to reduce the neural network complexity [22]. The dropout layer deactivates some of neurons in the training process. In this work, we have integrated a dropout layer between the two GRU layers to facilitate and accelerate the training step.

#### 3.6.3. Training and Testing Dataset

**Figure 8:**
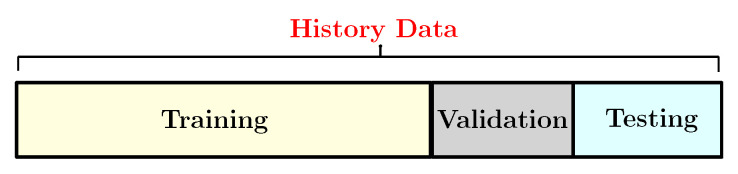
The specific category of the history data.

The dataset has been divided into three groups: training, validation and test groups as shown in Figure 8. The training dataset is a dataset of examples used during the learning process and is used to fit the network parameters, such as the weights, and to determine the optimal combinations of variables that will generate a good forecasting model. The validation dataset is a sample of data held back from training the model used to give an estimate of model skill while tuning the model’s hyperparameters in order to avoid the overfitting. Finally, the test set is generally what is used to evaluate competing models. In this work, the training set consists of 80% of the whole dataset. So, we run our model with different ratios and then this percentage is selected because it produces the best accurate predicted values. Indeed, the prediction model has been developed based on the training group. The rest, which represent 20% of the whole dataset, have been allocated as the test set for the model evaluation. Generally, the RNN model has been excused under different ratios based on training and testing data such as 90:10, 80:20, 70:30, 10:90 etc. train/test splits. Then this model selects the best train-to-test for the prediction [23]. The choice of the ratio depends on several factors such as the architecture of the model, type of data and the horizon of prediction. We train the LSTM, the GRU and the Drop-GRU algorithms with 280, 600 and 750 hidden units for the prediction of, respectively, one day, three days and a week. So, the window size of input and output parameters depends on the time scale of the load forecasting. We also use the Adam technique as an optimizer in our study. The learning rate is set to 0.01 and it decays every 50 epochs. Several Dropouts can also lead to various results, so we choose the appropriate Dropout by the experimental test.

### 3.7. Performance Evaluation Indicators

Many factors can influence the accuracy of prediction, including the authenticity of the data sources, the prediction techniques, the experimental conditions, etc. It is therefore necessary to use general indicators to evaluate the forecasting quality. The accuracy indicator is the most critical one, which proves the quality of a forecasting model directly. For this study, we have adopted three metrics to evaluate the model. They are: RMSE (Root Mean Square Error) and MAE (Mean Absolute Error) and the ***R*** (the correlation coefficient), which represent three ways of a number of approaches of comparing forecasts with their eventual outcomes. The formulations of these metrics are defined as [24,25]:

(21) $$\mathrm{RMSE}=\sqrt{\frac{1}{N}\sum_{i=1}^{n}{\left(y_{i}-\tilde{y_{i}}\right)}^{2}}$$

(22) $$\mathrm{MAE}=\frac{\sum_{i=1}^{n}\left|y_{i}-\tilde{y_{i}}\right|}{N}$$

(23) $$R=\frac{cov(y_{i},\tilde{y_{i}})}{\sigma_{y_{i}}\sigma_{\tilde{y_{i}}}},$$

where $y_{i}$ is the real value; $\tilde{y_{i}}$ is the prediction result of $y_{i}$ and *N* is the total number of testing samples. $cov(y_{i},\tilde{y_{i}})$ is the covariance of the two variables and $\sigma_{y_{i}}$, $\sigma_{\tilde{y_{i}}}$ represent the standard deviations of $y_{i}$ and $\tilde{y_{i}}$, respectively.

## 4. Experimental Results

In this work, we present a prediction of the power consumption of a survey area for different prediction horizons of “one day”, “three days”, “one week” and “two weeks”. In the proposed methodology, we have implemented three models, namely LSTM, GRU and Drop-GRU. For the several architecture structures of the various models, the network performance is dissimilar. The internal architecture of the proposed models is predefined and unchangeable; each topology admits a vector input of *n* values which are the current power consumption at time t=0 and the previous consumptions. We construct four neural networks of different architectures; each one is adapted to its prediction horizon. These networks are able to predict consumption after 30 min. Through the repetition process and the right choice of parameters for these networks, we can predict the entire period of the desired horizon. The parameters of each network (number of inputs, number of neurons in the hidden layer, number of iterations, number of outputs, …) are determined by the process of training on real data. The choice of the number of data for training is set at four times greater than the prediction horizon. The choice of these parameters is illustrated in Table 2.

As shown in this table, the neural network architecture depends on the number of days to predict. Indeed, the number of inputs, the number of units in the hidden layer and the size of training data are proportional to the prediction horizon.

### 4.1. Training and Validation Processes

**Figure 9:**
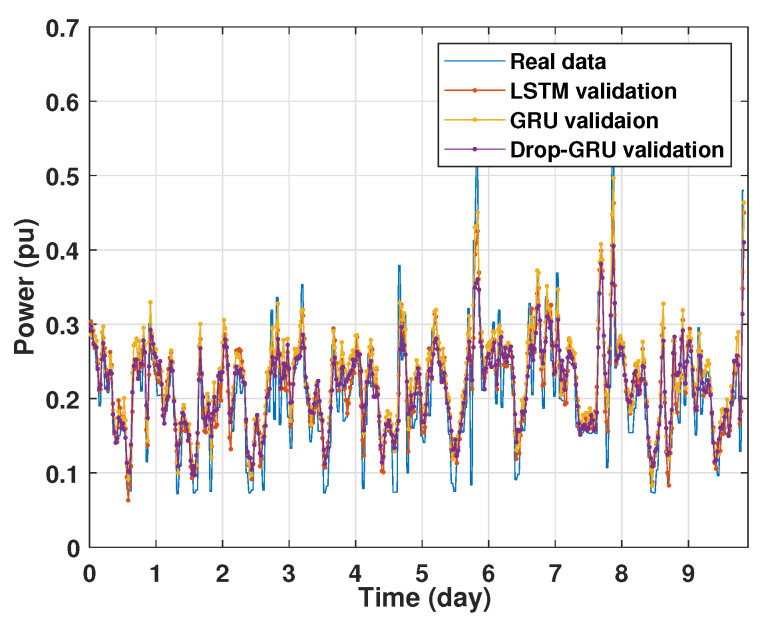
Training and validation for 1 day forecasting.

**Figure 10:**
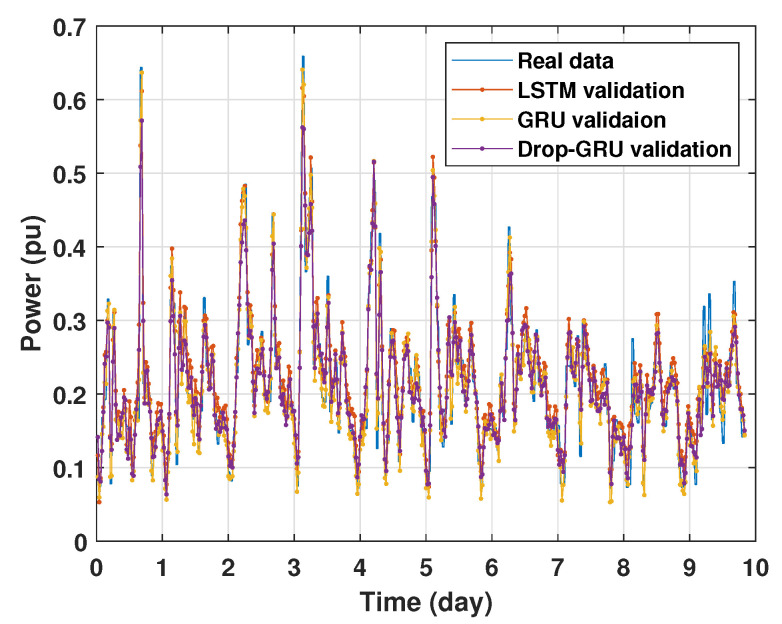
Training and validation for 3 days forecasting.

**Figure 11:**
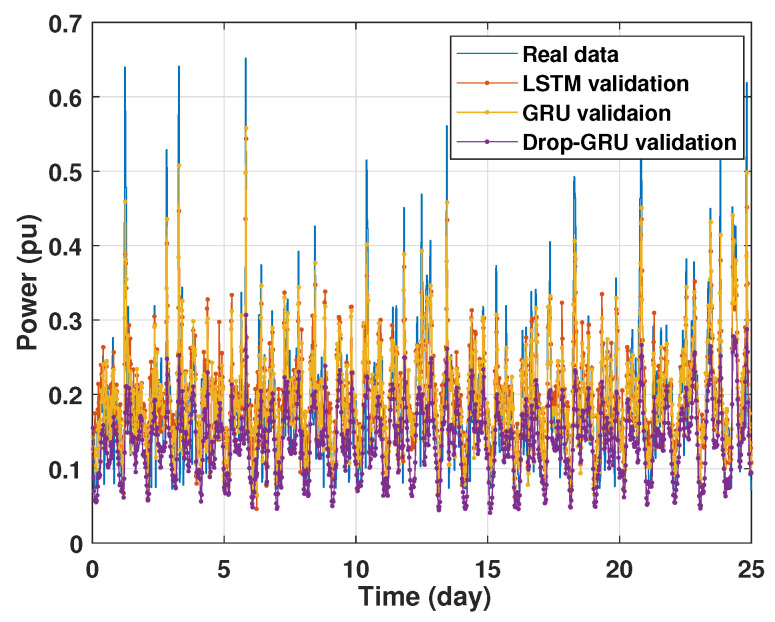
Training and validation for 7 days forecasting.

**Figure 12:**
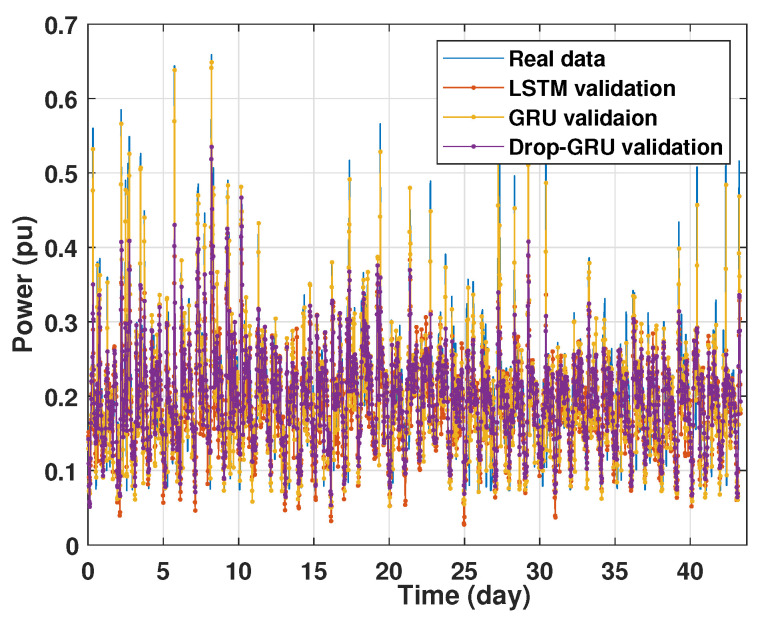
Training and validation for 15 days forecasting.

Machine learning models are usually evaluated by the validation process to measure the interpolation power. Figure 9, Figure 10, Figure 11 and Figure 12 illustrate this process. As shown, the validated curve of each neural network algorithm follows the trend of the original curve samples at a great percentage. Thus, only very small errors are seen in the validation of each approach which prove the performance of the model’s architecture.

### 4.2. Prediction of Power Consumption

**Figure 13:**
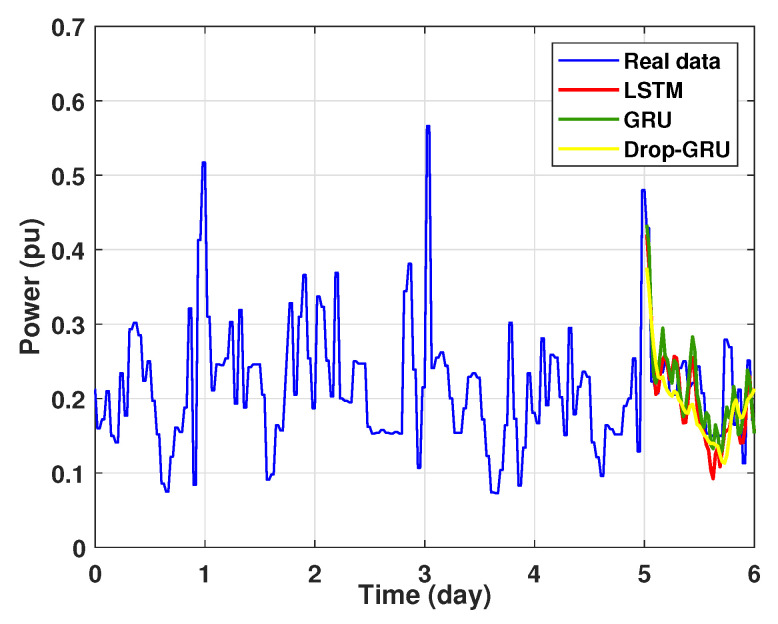
Prediction results of 1 day power consumption.

**Figure 14:**
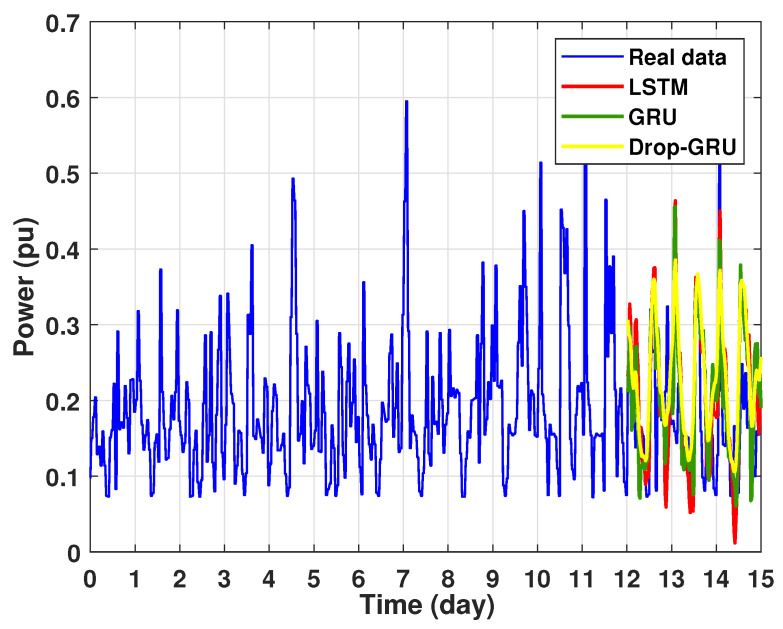
Prediction results of 3 days power consumption.

**Figure 15:**
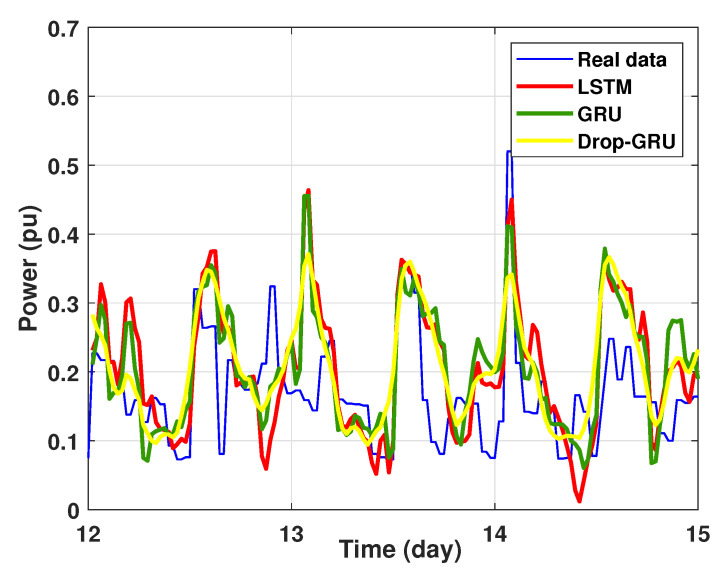
Zoom version Prediction of 3 days power consumption.

**Figure 16:**
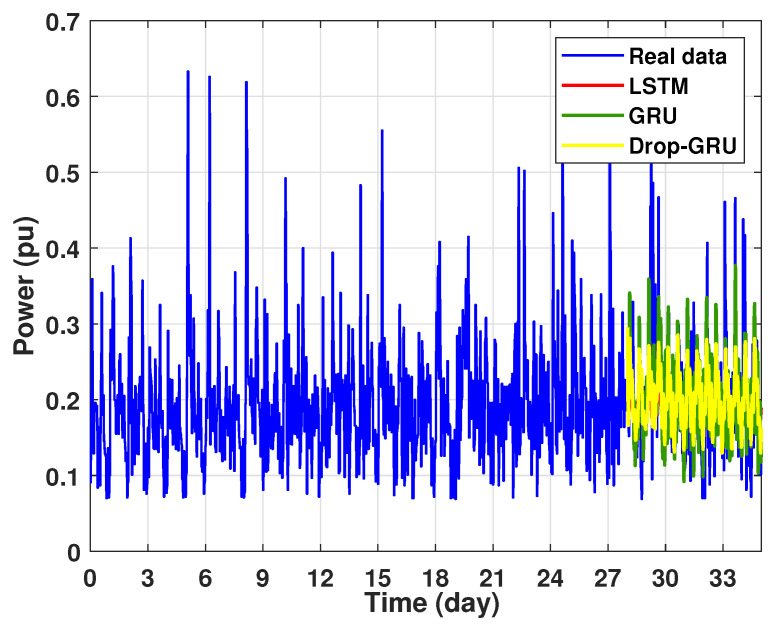
Prediction results of 7 days power consumption for the first area.

**Figure 17:**
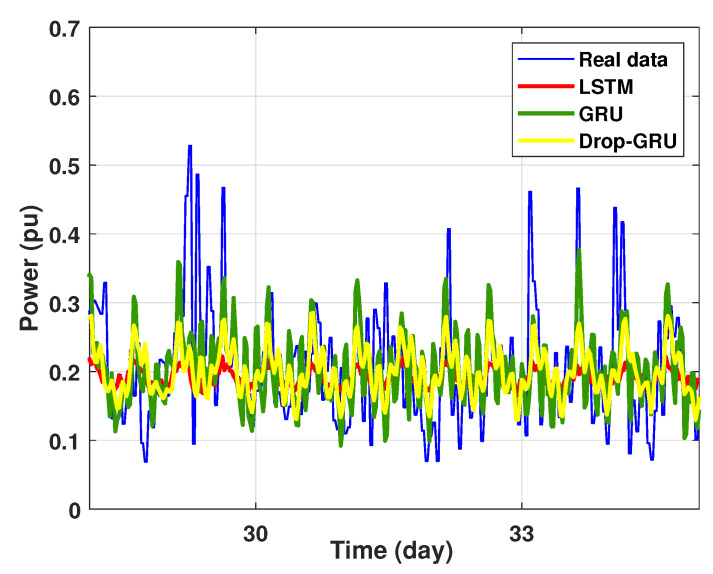
Zoom version Prediction of 7 days power consumption for the first area.

**Figure 18:**
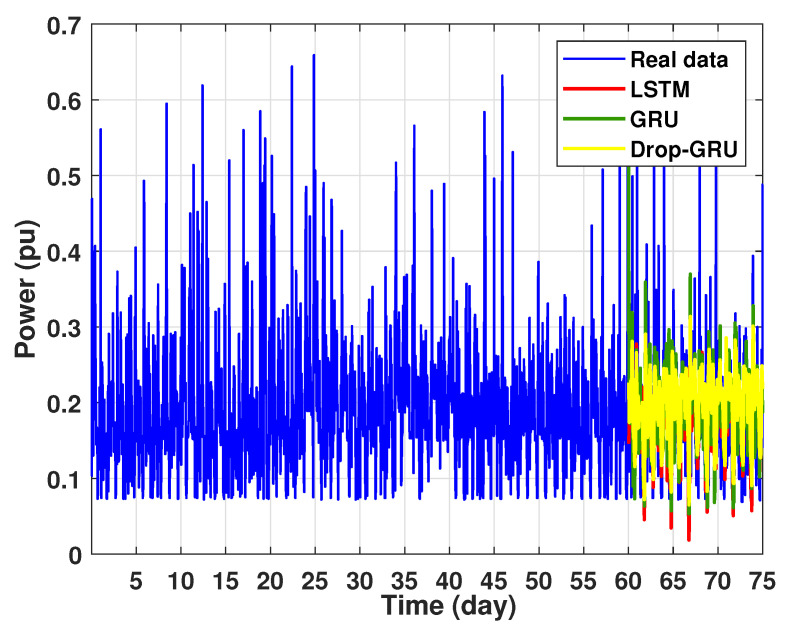
Prediction of 15 days power consumption for the first area.

**Figure 19:**
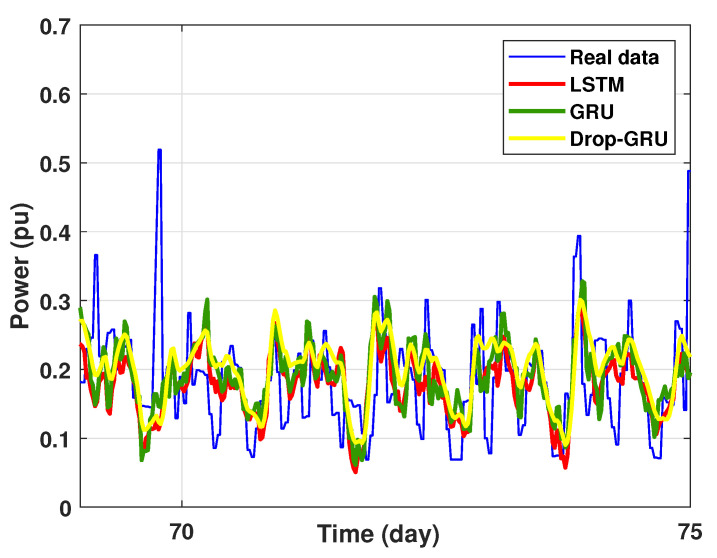
Zoom version Prediction of 15 days power consumption for the first area.

The graph for each experiment prediction model against the actual values is given in the Figure 13, Figure 14, Figure 15, Figure 16, Figure 17, Figure 18 and Figure 19 below.

- Experiment 1: 1 Day prediction

In the first experiment, we present the power consumption prediction of a studied area for one day. Figure 13 illustrates the prediction results of the three methods—LSTM, GRU and Drop-GRU. These results are similar to the true data value.

- Experiment 2: 3 Days prediction

The second experiment represents the power forecasting results over 3 days. Figure 14 shows the results prediction of the proposed algorithms. Figure 15 represents the zoomed version of these prediction results. It seems clear that the prediction curves keep the shape of the actual data curve and the three models, LSTM, GRU and Drop-GRU, are able to forecast the peaks of power consumption.

- Experiment 3: 7 Days prediction

In this experiment, a weekly prediction of the power consumption for the chosen area is presented in Figure 16 and the zoomed versions is illustrated in Figure 17. As shown, the prediction results detect the peaks of the power consumption at the same time as the actual data, but the prediction values of the numerous peaks are lower than the actual values. It can also be noted that the GRU model is the best one in the forecast of high values of power consumption.

- Experiment 4: 15 Days prediction

This last experiment gives the forecasting power results for the horizon time of 15 days. Figure 18 and Figure 19 show the prediction results of the three techniques. These experimental results illustrate the performance prediction of these models, especially in the detection of power peaks compared with the previous experiments.

### 4.3. Detection of Power Consumption Peaks and Load Shedding

**Figure 20:**
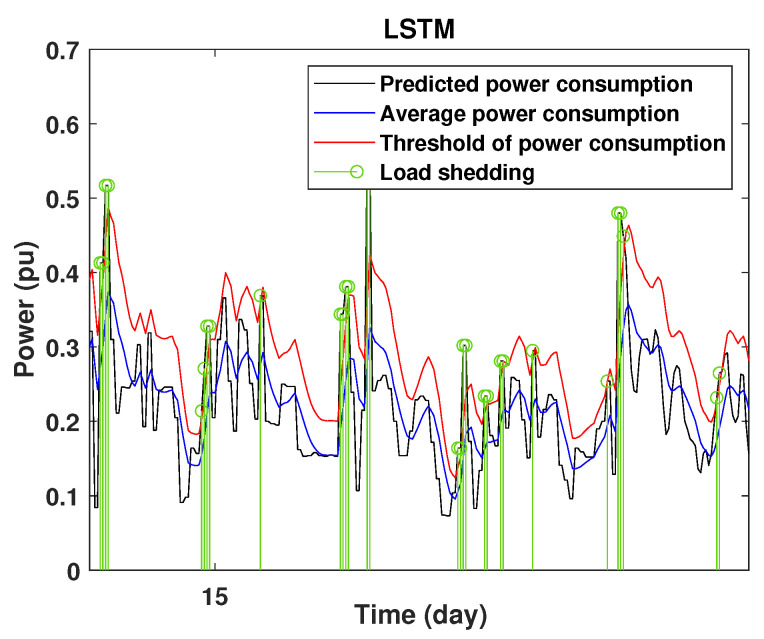
Detection of power consumption peaks during 1 day using the LSTM model.

**Figure 21:**
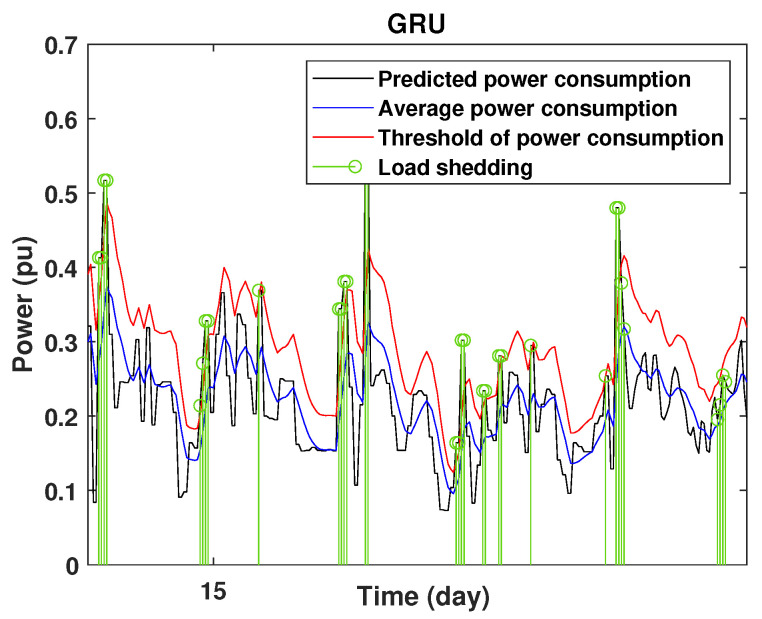
Detection of power consumption peaks during 1 days using the GRU model.

**Figure 22:**
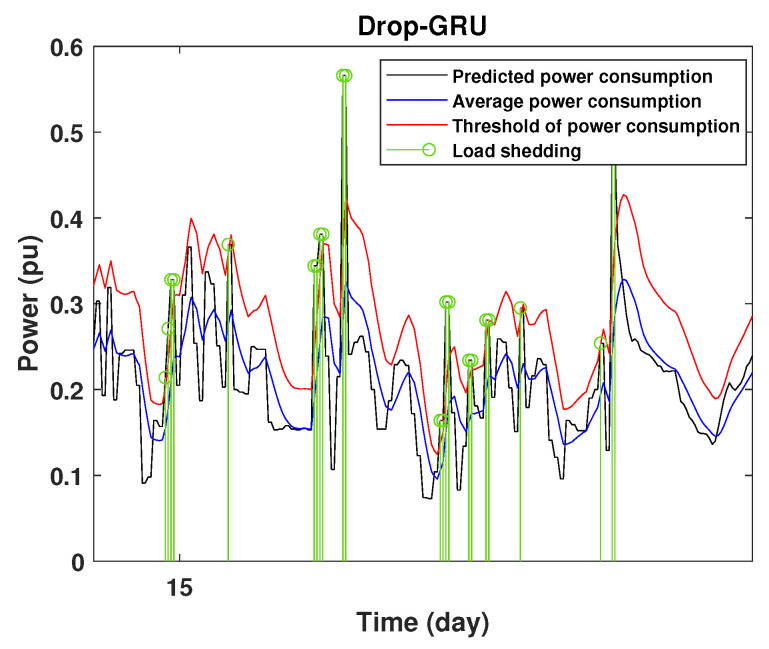
Detection of power consumption peaks during 1 days using the Drop-GRU model.

**Figure 23:**
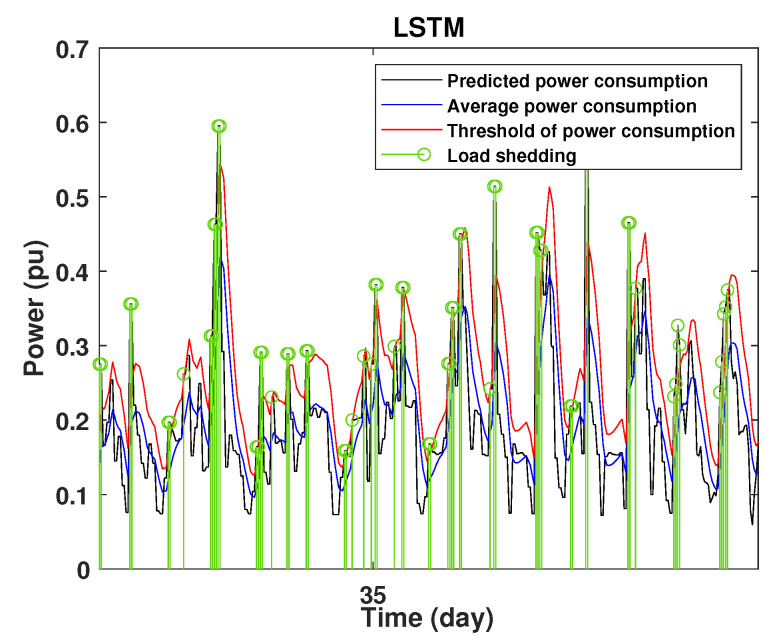
Detection of power consumption peaks during 3 days using the LSTM model.

**Figure 24:**
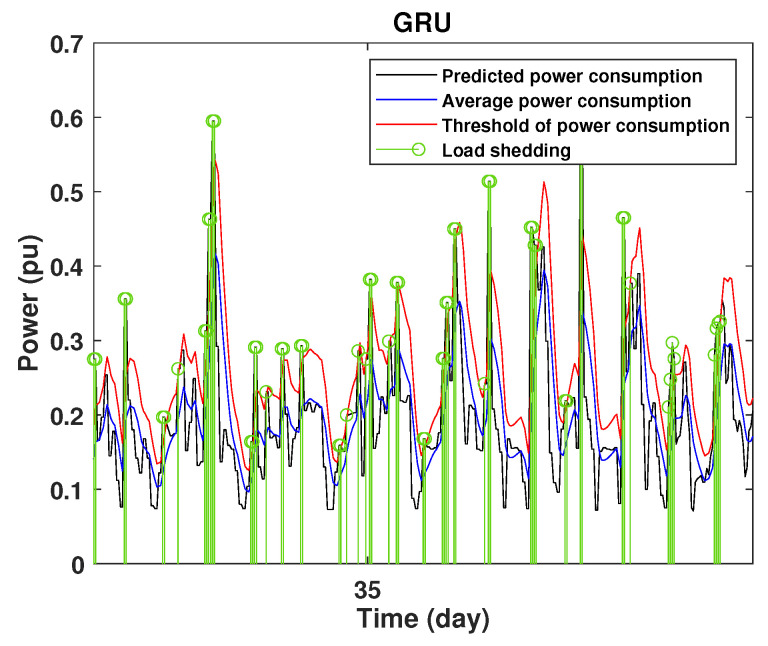
Detection of power consumption peaks during 3 days using the GRU model.

**Figure 25:**
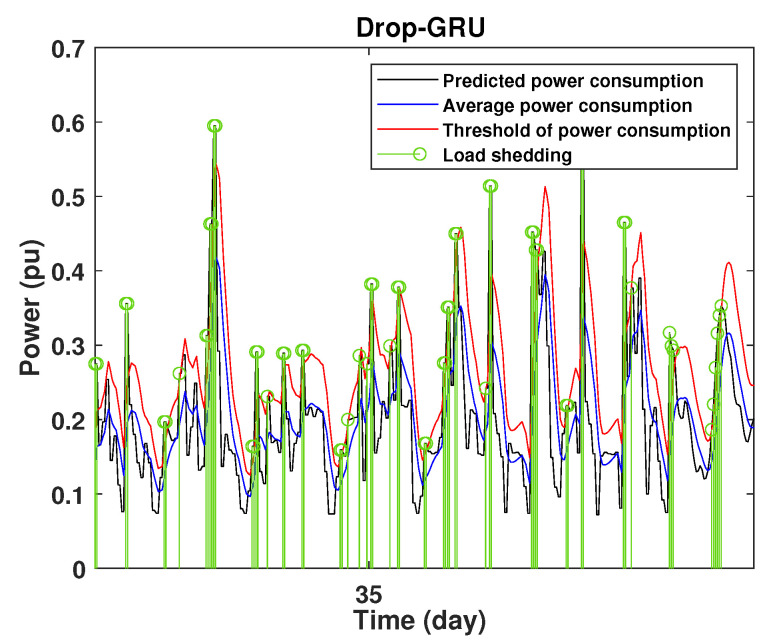
Detection of power consumption peaks during 3 days using the Drop-GRU model.

To define the instant at which the load should be disconnected, we determine the moving mean power consumption and we consider that the maximum power consumption should not exceed 15% of the average power. So, if the predicted power consumption exceeds the predefined value, a peak is detected and then the load is disconnected. Figure 20, Figure 21, Figure 22, Figure 23, Figure 24 and Figure 25 illustrate the detection of power consumption peaks.

As shown from these figures, the three models are able to predict the peaks of the power consumption with a very low shift of time compared to the real time. The power consumption forecasted by the GRU and Drop-GRU are very similar to the true data but the GRU model is better at the prediction of high values.

### 4.4. Analysis of Results

In this work, we mainly use three metrics to evaluate the performance of the proposed approaches on two aspects: accuracy and running time. Table 3 illustrates the results for the accuracy measurement and the execution time in seconds.

In this table, it is observed that the uncertainty of the predicted results of the three models and the execution times increase with the increasing of the prediction horizon. The statistics of simulation results of the LSTM, GRU and Drop-GRU models for the testing dataset show that the four RMSE and the four MAE values of LSTM models are larger than those of the GRU and GRU-Drop models. In contrast, the four correlation values *R* are smaller than those of GRU and Drop-GRU. Three of four correlation values of the Drop-GRU models are also larger than those of the GRU models.

The results show that the Drop-GRU neural network is more efficient and performs better than the GRU and the LSTM models. The Drop-GRU algorithm produces better results in terms of accuracy and prediction speed compared to the LSTM and GRU models. The GRU performed better than the Drop-GRU in the detection of high values of power consumption but it is less fast. We can deduce that the Drop-GRU architecture produces very satisfactory results and the prediction results are precise and reliable. The two performance indices, RMSE and MAE, have low values and *R* has perfect values (near +1), as shown in Table 3 and in the different prediction curves (Figure 13, Figure 14, Figure 15, Figure 16, Figure 17, Figure 18, Figure 19, Figure 20, Figure 21, Figure 22 and Figure 23). The consumption forecasts are close and representative of the actual consumption. We emphasize that the learning time depends on the approach used for forecasting. This learning time is proportional to the size of the network and to the prediction horizon.

To conclude, the three models are able to simulate power prediction processes accurately by employing time sequences input. The experimental results show the effectiveness of the proposed prediction algorithms for medium-short time period power load forecasting. GRU and LSTM models predict power consumption with good accuracy. Given that GRU has simpler structures and fewer parameters, and requires less time for model training, it may be the preferred method for short term prediction and it can be improved by hybridization with other techniques such as the dropout.

## 5. Conclusions

This paper presents an energy management strategy based on the forecasting process and proposes three deep neural networks: LSTM, GRU and Drop-GRU. The main objective of these approaches is to forecast and control the load consumption. The power consumption prediction methods firstly treated the input data, performed effective feature extraction and then built the appropriate network structure to optimize the ability prediction. Finally, a comparative study of the proposed algorithms is performed. These three techniques were implemented and tested on a set of power load data and the results indicated that the Drop-GRU was superior to the GRU and the LSTM. More specifically, the GRU approach is very suitable for our project to forecast the energy consumption over a defined horizon based on previous consumption readings, which will allow us to predict consumption peaks and predict in advance an optimal decision-making scenario for load shedding. The future direction of the research is to develop hybrid models with an even higher accuracy and even higher speeds and we can further improve these results by taking into account other external factors such as meteorological information and information on holidays. So, from the prediction results and the external data on the production capacity, it is easy to detect high consumption points that exceed the authorized consumption threshold and then protect the electrical grid. 

## Figures and Tables

**Table 1:** Trainable Weights in the LSTM network.

4np	4pp	4p	mp	*m*
The weights between the input layer and the hidden layer.	Recursive weights in the hidden layer.	The bias of the hidden layer.	The weights between the hidden layer and the output layer.	The bias of the output layer.

**Table 2:** LSTM forecasting architecture.

Number of Days to Predict	1 Day	3 Days	7 Days	15 Days
Data size (measure)	288	720	1680	3600
Number of training data	240	576	1344	1880
Number of data to predict	48	144	336	720
Number of units (LSTM /GRU) in the hidden layer (h)	280	600	750	1000
Number of inputs for the LSTM/GRU network (n)	200	300	300	600
Number of outputs for the LSTM/GRU network (m)	1	1	1	1
Number of trainable weights (*NTW*) for the LSTM network	539,001	2,163,001	3,153,751	6,405,001
Number of iterations	100	100	100	200

**Table 3:** Performance criteria of the studied area.

Algorithm	Evaluation Indices	Number of Days to Predict			
		1 Day	3 Days	7 Days	15 Days
LSTM	RMSE	0.0508	0.0904	0.0844	0.0837
	MAE	0.0399	0.0682	0.0606	0.0583
	*R*	0.9666	0.8716	0.8045	07337
	Execution time (s)	4.8058	9.1943	11.67130	43.8825
GRU	RMSE	0.0466	0.0868	0.0823	0.0873
	MAE	0.0381	0.0678	0.0616	0.0621
	*R*	0.9708	0.8781	0.8155	0.7067
	Execution time (s)	4.0829	7.7684	9.6836	33.6734
Drop-GRU	RMSE	0.0472	0.0727	0.0866	0.0813
	MAE	0.0363	0.0555	0.0612	0.0574
	*R*	0.9696	0.9097	0.8287	0.7482
	Execution time (s)	4.1069	7.7501	9.5361	33.5519

## Data Availability

Not applicable.
